# 

**Published:** 1893-03

**Authors:** 


### CONTENTS.

COMMUNICATIONS.

Progress and Needs of Dentistry........ 105
The Condition of the Dentine in Pulpless Teeth........ 115
Personal Experiences in Empyema of the Maxillary Sinus........ 124
Arrest of the Development and Decalcification of the Enamel of Dentine........ 130
Local Anæsthetics........ 143

SELECTIONS.

The Sleeping Habit........ 129
Compound Fracture of the Inferior Maxilla treated by Wire Suture 149

NOTICES.

The Pan-American Medical Congress........ 151
Special Notice........ 152
The St. Louis Dental Society........ 152

EDITORIAL.

Prospectus of the World's Columbian Dental Congress........ 153
Edgar Park, D.D.S........ 153
Obituary........ 156

